# An augmented patient-specific approach to administration of contrast agent for CT renal angiography

**DOI:** 10.1590/S1677-5538.IBJU.2018.0366

**Published:** 2019-01-29

**Authors:** Charbel Saade, Nadine Hamieh, Ibrahim Al-Sheikh Deeb, Maurice Haddad, Alain S. Abi-Ghanem, Diamond Ghieh, Fadi El-Merhi

**Affiliations:** 1 Department of Radiology American University of Beirut Beirut Lebanon Department of Radiology, American University of Beirut, Beirut, Lebanon

**Keywords:** Computed Tomography Angiography, Kidney, Radiation Dosage

## Abstract

**Purpose:**

This hybrid retrospective and prospective study performed on 200 consecutive patients undergoing renal CTA, investigates the opacification of renal vasculature, radiation dose, and reader confidence.

**Materials and Methods:**

100 patients were assigned retrospectively to protocol A and the other 100 were allocated prospectively to protocol B. Both protocols implemented a contrast material and saline flow rate of 4.5 mL/sec. Protocol A utilized a 100 mL of low-osmolar nonionic IV contrast material (Ioversol 350 mg I/mL) while protocol B employed a patient-tailored contrast media formula using iso-osmolar non-ionic (Iodixanol 320 mg I/mL).

**Results:**

Arterial opacification in the abdominal aorta and in the bilateral main proximal renal arteries demonstrated no statistical significance (p>0.05). Only the main distal renal artery of the left kidney in protocol B was statistically significant (p<0.046). In the venous circulation, the IVC demonstrated a significant reduction in opacification in protocol B (59.39 HU ± 19.39) compared to A (87.74 HU ± 34.06) (p<0.001). Mean CNR for protocol A (22.68 HU ± 13.72) was significantly higher than that of protocol B (14.75 HU ± 5.76 p< 0.0001). Effective dose was significantly reduced in protocol B (2.46 ± 0.74 mSv) compared to A (3.07 ± 0.68 mSv) (p<0.001). Mean contrast media volume was reduced in protocol B (44.56 ± 14.32 mL) with lower iodine concentration. ROC analysis demonstrated significantly higher area under the ROC curve for protocol B (p< 0.0001), with inter-reader agreement increasing from moderate to excellent in renal arterial visualization.

**Conclusion:**

Employing a patient-tailored contrast media injection protocol shows a significant refinement in the visualization of renal vasculature and reader confidence during renal CTA.

## INTRODUCTION

CT Angiography (CTA) is established as one of the noninvasive imaging modalities for the evaluation of vascular diseases. Since its development, Renal CTA (rCTA) has emerged as a reliable tool for the diagnosis of renal artery stenosis. The sensitivity and specificity of rCTA for the diagnosis of greater than 50% renal artery stenosis range from 67%-100% and 77%-98%, respectively (1). On the other hand, renal magnetic resonance angiography (MRA) has sensitivity and specificity of 88%–100% and 70%–100% with low interobserver variability, especially for severe stenosis greater than 70% (2, 3). However, the sensitivity and specificity are dependent upon the opacification levels in the renal vasculature. Over the years, improvements in CTA to evaluate renal artery stenosis have resulted from optimization of acquisition (4, 5), image presentation with various rendering algorithms, as well as contrast media administration protocols. Recent studies have reported attenuation values of the renal arteries being as high as 435±48 HU, while those of the renal veins have reached 277±29 HU (6), whilst employing large contrast media volumes (60-125 mL) (5-8).

There are three main approaches in determining contrast media volume. The first approach is body weight range and fixed contrast volume-based protocols; 80 mL for <61 Kg, 90 mL for 61-91 Kg and 120 mL for > 91 kg (9). The second is linear body weight and contrast volume 1-1.5 mL/kg (10) and finally; fixed contrast volumes ranging from 60 to 125 mL (11). Furthermore, previous studies have reported that weight-based protocols are not considered to be a determining factor during CTA (3, 12-14). The aim of our study is to investigate the opacification of renal vasculature, radiation dose, and reader confidence by a patient-tailored contrast administration protocol during rCTA.

## MATERIALS AND METHODS

### Patient Selection

This hybrid retrospective and prospective study was approved by the institutional review board. Written informed consents were only waived for protocol A, whilst, informed consents were mandatory and obtained for protocol B. Two hundred rCTA were evaluated from July 2012 to September 2015. Between July 2012 and June 2014, one hundred patients with suspected renovascular disease went through the conventional CTA contrast protocol (protocol A). Between July 2014 and September 2015, the other one hundred underwent the patient-tailored contrast material injection protocol (protocol B) (Table-1). Patients were distributed normally. Patients with serum creatinine >1.2 mg/dL or eGFR <60 mL/min/1.73 m^2^, and pregnant patients were excluded from the final patient cohort (n=8).

**Table 1:** Demographics.

	Protocol A	Protocol B
	
Gender		
Females	30	33
Males	70	68
Age	58.49±19.09	52.09±16.05
Height	1.70±0.90	1.71±0.09
Weight	78.11±15.37	78.91±14.78
BMI	26.96±4.75	27±4.67

### Renal CT Angiography Acquisition

All examinations were done using a 256-slice MDCT scanner (Brilliance iCT); patients were placed in supine position. Before the scan acquisition, anterior-posterior scout scan was performed, with a scan range from the diaphragm to the iliac crest. CT scan parameters employed in both protocols were: detector width of 256×0.625 mm, pitch of 0.881:1 ratio, and rotation time of 0.27 sec, 120 kVp, effective 180 mAs, with x,y and z-axis modulation (DoseRight), and hybrid iterative reconstruction iDose4, level 5.

### Bolus triggering technique

The two protocols used distinct bolus-tracking techniques. Protocol A harnessed a dynamic bolus tracking: the region of interest (ROI) is marked in the lumen of the suprarenal segment of the abdominal aorta with a constant contrast volume of 100 mL. 100 HU was chosen as a trigger attenuation value threshold above the baseline with a delay of 5s (upon reaching the peak threshold to the beginning of the CTA acquisition). Each bolus employed free breathing and scanner parameters; rotation time of 0.5 sec, 100 kVp, effective 50 mAs and interscan delay 1 sec. The volume of contrast was based on current departmental work practice and in line with current literature (3, 12, 16, 17), it was not adjusted to the patient’s body mass index (15). Protocol B harnessed the test bolus technique: the ROI is marked within the abdominal aorta (suprarenal segment) using a small amount of contrast (5 mL), which is not part of the total contrast volume (CV) measured using the formula. It is administered at the same rate, as we measured the time to peak (TTP) and the main bolus. Both protocols employed a 100 mL saline chaser injected at 4.5 mL/s.

### Contrast Medium Administration

An automated dual barrel power injector (Optivantage®) was used to inject warmed contrast material (37º) through a 20 gauge venous catheter in the right arm. Patients were examined by two contrast media protocols. Protocol A, conventional protocol consisting of a 100 mL of contrast (Ioversol 350 mg I/mL) injected intravenously at a flow rate of 4.5 mL/s. Protocol B utilized a patient-specific contrast media formula: CV = (ST + TTP - OVWP) x FR (Iodixanol 320 mg I/mL). ST: scan time; TTP: time to peak of the contrast at the level of the renal arteries; OVWP: optimal venous washout phase (12 seconds) (3, 12, 16); FR: flow rate. Both protocols employed 100 mL saline at 4.5 mL/s. Two separate iodinated contrast media agents were chosen to reduce the iodine concentration administered to patients with the patient-specific contrast media formula.

### Radiation Dose Measurement

The dose-length products (DLP [mGy × cm]) were recorded from the patient protocol, then individual effective dose (E[mSv]) was calculated from the DLP for each of the CT scans (17). In order to calculate the E_,_ a normalized conversion factor (k[mSv / mGy × cm]) for the abdomen —0.015 mSv/mGy × cm— was used (18): E=DLP x k.

### Image assessment

With a smooth convolution kernel (field of view 380 × 380 mm, image matrix, 512 × 512), we reconstructed trans-axial images with 1.5 mm slice thickness (1 mm increment). Our department’s CT experts (CS, 15 years) determined the technical inclusion criteria, to ensure a correct scan range, as well as an anatomical inclusion of the pathway, origin and termination of the renal vasculature for each of the prospective and retrospective cases. Using a primary reporting workstation (IMPAX 6.3.1, AGFA) with a GSDF-calibrated 3-megapixel monitor, quantitative measurements of all images were performed.

### Vascular Opacification Analysis

**Figure 1:**
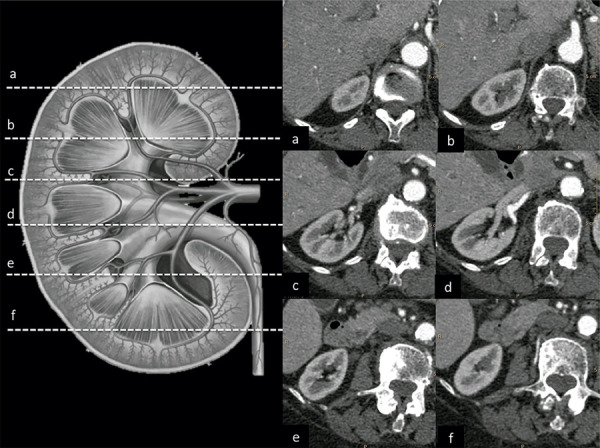
Anatomical location of measurements of the renal vasculature. The segmental lines are as follows: (a and b) upper pole of the kidney that demonstrates the renal cortex, medulla and renal pyramids as well as the minor calyx and interlobular arteries; (c and d) renal cortex, medulla, renal pyramids, interlobular and main segmental renal arteries; and (e and f) inferior pole of the kidney that shows the renal cortex, medulla and renal pyramids as well as the minor calyx and interlobular arteries.

A circular ROI diameter was fitted within the lumen of the vessel, and opacification was measured in the axial plane within it in Hounsfield units (HU). Then, within the ROI of each vessel, the mean and standard deviation (SD) were recorded. In the pre-contrast and arterial phase, both arterial and venous structures were measured. Arterial measurements of the abdominal aorta, the main segments (proximal and distal) of bilateral renal arteries, and the interlobular segments (superior and inferior) of bilateral renal arteries were determined. Venous measurements included the inferior vena cava (IVC), right and left renal veins in both the proximal and distal segments (Figure-1).

### Contrast-to-Noise Ratio Measurement

Using a 1.5 mm thick trans-axial image, we calculated the contrast to noise ratio (CNR).

Image quality depends on several factors among which are noise, resolution, and mechanical along with electrical stability of the instrument in use. Noise is not an independent factor as it always depends on the clarity of the available information, therefore it is to be correlated with the contrast in the image under study. The CNR serves as a quantitative assessment tool for noise relative to the signal between high and low density structures.

The ROI was drawn at the same size of the vessel lumen diameter, avoiding soft and/or calcified plaques of the vessel wall. When calculating the CNR, we measured the attenuation of the right psoas muscle (ROI_PSM_) at the level of the 1st Lumbar vertebrae, followed by the second measurement of noise as the standard deviation. The mean opacification of each patient was measured at the origin of the renal arteries (ROI_RA_) in order to compare the overall degree of vascular opacification within the renal vasculature. Finally, the CNR was calculated based on the measured parameters described above with an empirically derived formula;


$$\mathrm{CNR}\;=\;\left({\mathrm{ROI}}_{\mathrm{RA}}-{\mathrm{ROI}}_{\mathrm{PSM}}\right)/\mathrm{Noise}$$

### Diagnostic Efficacy

Thirty two (n=32) cases were chosen with an equal number of normal (n=16) and abnormal cases (n=16), for each contrast protocol (total=64). The normal cases showed normal renal vasculature, while the abnormal cases showed varying degrees of atherosclerotic changes, as defined by the radiologists’ reports. Images were selected by one of our department’s experts in CT imaging, and not involved in the image reading protocol. Readers viewed images in a blinded manner and in a single sitting. All pathology was visible on the trans-axial images. Vascular pathology prevalence in the image bank was not disclosed to the readers.

Three radiologists with a mean of *17 years’ experience (F.M 14 years, M.H 35 years and A.A 4 years)* certified by the American Board of Radiology and *The Royal College of Radiologists*, were the base of the multi-reader analysis. Manipulating the level of the images and the window was permitted to the readers. Each reader indicated the locations of suspicious findings with a confidence level noted from 1-5 where 5 indicated a definite presence of vascular pathology whereas 1 indicated pathology was definitely not present.

### Visual Grading Assessment

In order to illustrate viewer preference of one technique over another (based on the visibility of the renal vasculature), the visual grading characteristic (VGC) method (19) was used. VGC is widely used to assess for clinical image quality in radiography, where the observer rates his confidence with the image quality depending on whether or not it has met the image quality criteria. For this study, confidence level ranged from 1-5 where 5 indicated excellent renal artery visualization and 1 represented poor renal artery visualization.

### Statistical analysis

Data entry and statistical analyses were performed using SPSS, V.23, 2009. Descriptive analyses for age distribution between the two patient groups was carried out by reporting frequencies and percentages. Continuous variables were presented: means and standard deviations were calculated using independent-samples t-test to compare: age, anteroposterior and transverse diameter, abdominal circumference, contrast media volume, dose length product, radiation dose, and measured opacity between the two patient groups.

To account for the potential confounding effect of the abdominal circumference on the decrease in radiation dose, multivariate logistic regression analyses were carried out. This was also controlled by stratified analyses adjusting our population into four subgroups according to patients’ abdominal circumference range, and the usage of independent t-tests to compare the variables.

The Dorfman-Berbaum-Metz approach was employed in order to do the ROC analyses. This approach uses cases as fixed and readers as random. Cases were treated as fixed on the basis that the limited image sample size was not taken as a representative of all images. Cohen’s kappa analysis was used to calculate. Inter-observer agreements were calculated using *k* values of 0.60-1, 0.41-0.60, 0.21-0.40, and <0.20 that defined excellent, moderate, fair, and poor agreement respectively. Results were considered statistically significant if p≤0.05.

## RESULTS

### Vascular Measurements and CNR

**Figure 2:**
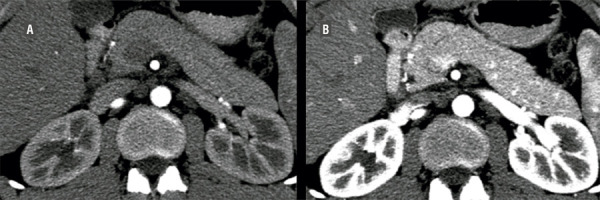
Demonstrates contrast media timing technique for protocol A (b) and protocol B (a). Image a clearly displays only arterial opacification of the renal arteries as well as interlobular renal arteries, whereas image b demonstrates venous contamination within the renal collecting system as well as renal parenchyma.

Arterial measurements in the abdominal aorta demonstrated no statistical significance: protocol A=290.35 ± 105.82 vs Protocol B = 269.47 ± 58.74 (p=0.086) (Figure-2). In both protocols, the right and left main proximal renal arteries demonstrated no statistical significance (p>0.05). As for the distal segments of the renal arteries, only that of the left kidney in protocol B was statistically significant (p<0.046). The arterial opacification of the right and left interlobular arteries showed a clear difference in the two protocols (Table-2). In the venous circulation, the IVC demonstrated a significant reduction of opacification in protocol B (59.39 ± 19.39) compared to A (87.74 ± 34.06) (p<0.001). Also, protocol B demonstrated a significant reduction in venous opacification of the proximal and distal segments of bilateral renal veins comparing to protocol A (p<0.001) (Table-2). Mean CNR for protocol A (22.68 HU ± 13.72) was significantly higher than that for protocol B; (14.75 HU ± 5.76 P<0.0001).

**Table 2:** Opacification measurements of the renal vasculature.

Vascular Measurements	Right Kidney			Left Kidney		

	Protocol A	Protocol B	*P*	Protocol A	Protocol B	*P*
**Arterial**						
**Main Renal artery**						
Proximal	261.45 ± 99.30	255.76 ± 52.49	0.613	267.74±101.17	250.38 ± 56.44	0.136
Distal	254.47 ± 91.95	238.83 ± 55.23	0.146	253.45 ± 88.77	232.34 ± 56.19	0.046
**Interlobular**						
Superior	196.31 ± 42.16	200.67 ± 54.60	0.527	197.27 ± 42.73	204.63 ± 54.91	0.291
Inferior	184.99 ± 43.99	206.34 ± 60.55	0.005	182.10 ± 38.34	210.27 ± 64.53	0.001

**Venous**						
**Renal Vein**						
Proximal	123.03 ± 61.11	78.49 ± 38.43	0.001	116.84 ± 53.24	77.83 ± 39.59	0.001
Distal	121.77 ± 55.60	76.99 ± 37.44	0.001	120.99 ± 49.48	73.04 ± 34.75	0.001

Data are mean ± standard deviation

### Renal parenchymal measurements

Renal parenchymal segmental measurements in the non-contrast phase demonstrated no significant differences in the upper, middle, and lower segments of the cortex and medulla, except for the upper, middle, and lower medulla of the left kidney. In the arterial phase, the upper middle cortex, the lower cortex, and the medulla demonstrated significant differences between the two protocols (p<0.001), with Protocol B being lower than protocol A (Table-3).

**Table 3:** Opacification measurements of the renal parenchyma.

Renal Parenchyma	Right Kidney			Left Kidney		

	Protocol A	Protocol B	*P*	Protocol A	Protocol B	*P*
**Pre Contrast Phase**						
**Renal Pelvis**	14.01 ± 6.51	11.09 ± 4.56	0.001	12.81 ± 6.82	10.36 ± 4.88	0.004
**Cortex**						
Upper	32.51 ± 11.20	33.77 ± 4.58	0.301	32.67 ± 11.54	33.63 ± 4.65	0.442
Middle	34.71 ± 12.07	32.60 ± 4.07	0.100	33.26 ± 9.68	33.17 ± 4.66	0.936
Lower	31.39 ± 9.51	32.58 ± 4.34	0.285	33.65 ± 12.12	32.22 ± 4.13	0.266
**Medulla**						
Upper	30.60 ± 8.40	34.27 ± 5.52	0.001	31.16 ± 10.90	34.00 ± 4.57	0.017
Middle	32.00 ± 9.56	33.30 ± 5.23	0.223	31.12 ± 10.68	33.77 ± 4.69	0.025
Lower	31.39 ± 9.51	32.75 ± 4.67	0.200	30.68 ± 9.27	34.97 ± 4.93	0.001

**Arterial Phase**						
**Renal Pelvis**	26.29 ± 17.03	38.34 ±60.50	0.056	23.03 ± 17.48	36.81 ± 52.05	0.013
**Cortex**						
Upper	151.53 ± 48.92	100.28 ± 33.80	0.001	149.88 ± 38.82	100.18 ± 33.55	0.001
Middle	159.10 ± 48.04	105.30 ± 36.12	0.001	158.45 ± 41.92	102.94 ± 35.31	0.001
Lower	163.07 ± 50.08	108.28 ± 35.43	0.001	160.22 ± 45.22	103.04 ± 36.19	0.001
**Medulla**						
Upper	106.50 ± 55.92	48.76 ± 20.60	0.001	105.58 ± 58.23	48.84 ± 19.15	0.001
Middle	107.26 ± 58.98	51.47± 21.47	0.001	106.20 ± 60.28	50.00 ± 18.61	0.001
Lower	110.56 ± 58.24	51.86 ± 20.48	0.001	108.62 ± 58.44	50.64 ± 19.73	0.001

Data are mean ± standard deviation

### Contrast media volume

Contrast media volume was significantly reduced in protocol B (44.56 ± 14.32 mL) compared to A (100 ± 1.0 mL) (p<0.001), with the total CM calculation does not include the 5 mL contrast media test-bolus.

### Radiation Dose

Radiation dose was significantly decreased in protocol B (2.46 ± 0.74 mSv) compared to protocol A (3.07 ± 0.68 mSv) (p<0.001). To account for the potential confounding effect of the abdominal circumference on the decrease in radiation dose, stratified analyses were carried out. Our population was adjusted into three subgroups according to the patients’ abdominal circumference range, and radiation dose. Independent t-tests were used for comparison. Abdominal circumference was calculated after measurements of the anterior-posterior length and the transverse length, using this formula (20):


447Transverse Length/2^2+Anteriorposterior Length/2^22

In Table-4 summarizes the results of the stratified analyses and shows that radiation dose was still decreased in each of the subgroups. Furthermore, on multivariate analysis, radiation dose was also decreased in protocol B after adjustment for abdominal circumference (*r*=-0.634, p-value < 0.001).

**Table 4:** Stratified analyses for the radiation dose.

Abdominal Circumference (mm)	Number of Cases		Radiation Dose		P

	A	B	A	B	
<849	27	43	3.05 ± 0.61	2.49 ± 0.55	<0.001
>850-<940	29	37	3.04 ± 0.79	2.49 ± 0.91	0.012
>941	44	21	3.10 ± 0.65	2.33 ± 0.74	<0.001

Radiation dose: (mSv)

### Image Evaluation

**Figure 3:**
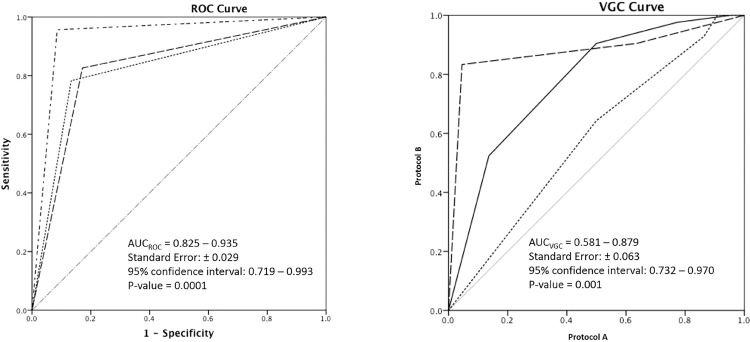
a) ROC curve and b) VGC curve. Each curve demonstrates the individual readers (lines) area under the curve at 95% confidence intervals. In both graphs there is statistical significance in area under the curve in protocol B compared to A.

Receiver operating characteristic - the five-point scale revealed a significant difference (p<0.005) between the two protocols with mean ROC values demonstrating increased reader confidence in protocol B compared to A with the area under the curve reaching 0.935 with reader confidence interval between 0.719 and 0.993 (Figure-3a).

**Visual grading characteristic** - the five-point scores were individually graded by the three readers for each protocol. The results were represented as a graph shown in Figure-3. When a preference is shown towards one protocol the curve is convex to that protocol’s axis. The graphs clearly demonstrate that when the renal arteries were assessed for opacification, the preference is for protocol B over A (Figure-3b).

**Kappa analysis** - rCTA yielded moderate interobserver agreement with protocol A (*k*=0.51) and B (*k*=0.73). There was a strong positive relationship between mean renal arterial opacification, good image quality, and reader confidence in protocol B compared to A (*r*=0.51, *p*<0.001).

## DISCUSSION

In the current study, we examined a patient-tailored contrast media protocol compared to the conventional contrast media injection protocol. We employed a multi-parametric model to perform the comparison, in which we considered opacification levels within blood vessels, CNR, and ROC analysis with the overall aim of investigating the effect of the protocol on the diagnosis of renovascular diseases. The results were consistent: The patient-tailored approach clearly reduced the opacification of the veins without compromising the arterial vasculature opacification, thus potentially reducing vascular artifact, however, there was increased noise in protocol B that resulted in a lower CNR, but, without affecting the subjective VGC. Interestingly, a reduction in iodine concentration in protocol A (350 mg/mL) compared to B (320 mg/mL) revealed that iodine concentration has no effect on vascular opacification since the emphasis is based on cardiovascular timing and contrast media volume control. Additionally, when observers are blinded in reading arterial studies, it was noticeable that the effect of venous contamination reduces the relative arterial opacification in Protocol A, however, when compared to protocol B, lower arterial opacification with significantly reduced venous contamination in the background gave rise to the observers’ perception that higher arterial opacification is best judged relative to low venous contamination. Hence, arterial opacification is determined based on the level of surrounding venous contamination which may distract observers when grading studies for their quality and was evident by greater reader confidence with narrower confidence intervals at 95% CI at lower iodine concentrations and vascular opacification of the renal vasculature. Expert radiologists demonstrated higher AUC values in protocol B compared to protocol A. The consistency of the improvement with the patient-tailored approach, regardless of the metric used, clearly accentuates the positive impact of our proposed technique.

Previous studies have shown a cost to achieving optimal image quality with rCTA examinations, in particular in regards to radiation dose (7, 21). Exceptionally, in our work, radiation dose was actually reduced with reduced contrast media volume, however, further work is required to validate this claim. This was due to the flying focal spot detecting changes in tissue attenuation throughout mA modulation by reduced contrast media within the parenchyma when administering patient-specific contrast media. This dose saving offers significant benefits to the examination since radiation levels at adjacent radiosensitive anatomical structures such as the adrenal glands and liver are reduced. The interplay between the radiation dose and contrast media protocols have often been overlooked, with the chief focus being on peri-venous artifact reduction via patient-specific contrast material formulas during CT angiography (3, 16), reduced x-ray tube voltage (22), and contrast media with low iodine concentration, while attempting to maintain image quality (23). The current study highlights the value of patient-tailored contrast media administration technique that can reduce radiation dose to patients during rCTA (irrespective of body habitus as proved after accounting for the potential confounding effect of the abdominal circumference). This decrease in CV and radiation comes at no cost since it is associated with increased image quality and reader confidence. Currently, it is somewhat difficult to draw an accurate comparison with the literature, and to our knowledge, we are the first to compare the patient-tailored to conventional contrast media approach for renal artery disease during rCTA.

There are limitations in this study; the use of conventional angiography could further clarify the diagnostic accuracy and patient outcome on the basis of our patient-tailored contrast media protocol. We did not test the same patients under both protocols. We did not compare renal arterial cross sections and luminal diameters with those of filtered back projection, hybrid, and model-based iterative reconstruction algorithms. Therefore, predicting accurate clinical outcomes in renal vasculature with our patient-tailored contrast media technique would ideally be confirmed with the use of conventional angiography and effects on clinical outcomes. Finally, we did not entertain the observer performance of image quality compared to the CNR, since observer performance employs noise texture (noise power spectrum) (24) when reducing radiation dose during iterative reconstruction.

In summary, we present a patient-tailored contrast media injection protocol that demonstrates significant improvements in the visualization of renal vasculature reader confidence during rCTA.

**Key points:**

1. Iodixanol improved visualization at reduced radiation dose during renal CT Angiography.
2. Iodixanol reduced the opacification of the veins without compromising the arterial vasculature.
3. When administering Iodixanol, radiation dose was reduced with reduced contrast media volume.
